# Bordonein-L, a new L-amino acid oxidase from *Crotalus durissus terrificus* snake venom: isolation, preliminary characterization and enzyme stability

**DOI:** 10.1186/s40409-015-0025-8

**Published:** 2015-08-13

**Authors:** Karla C. F. Bordon, Gisele A. Wiezel, Hamilton Cabral, Eliane C. Arantes

**Affiliations:** Department of Physics and Chemistry, School of Pharmaceutical Sciences of Ribeirão Preto, University of São Paulo (USP), Avenida do Café, s/n, Ribeirão Preto, 14040-903 SP Brazil; Department of Pharmaceutical Sciences, School of Pharmaceutical Sciences of Ribeirão Preto, University of São Paulo (USP), Ribeirão Preto, SP Brazil

**Keywords:** *Crotalus durissus terrificus*, L-amino acid oxidase, Rattlesnake, Enzyme activity, Enzyme stability, Chromatography, Snake venom, Yellow venom, Stabilization

## Abstract

**Background:**

*Crotalus durissus terrificus* venom (CdtV) is one of the most studied snake venoms in Brazil. Despite presenting several well known proteins, its L-amino acid oxidase (LAAO) has not been studied previously. This study aimed to isolate, characterize and evaluate the enzyme stability of bordonein-L, an LAAO from CdtV.

**Methods:**

The enzyme was isolated through cation exchange, gel filtration and affinity chromatography, followed by a reversed-phase fast protein liquid chromatography to confirm its purity. Subsequently, its N-terminal amino acid sequence was determined by Edman degradation. The enzyme activity and stability were evaluated by a microplate colorimetric assay and the molecular mass was estimated by SDS-PAGE using periodic acid-Schiff staining and determined by mass spectrometry.

**Results:**

The first 39 N-terminal amino acid residues exhibited high identity with other snake venom L-amino acid oxidases. Bordonein-L is a homodimer glycoprotein of approximately 101 kDa evaluated by gel filtration. Its monomer presents around 53 kDa estimated by SDS-PAGE and 58,702 Da determined by MALDI-TOF mass spectrometry. The enzyme exhibited maximum activity at pH 7.0 and lost about 50 % of its activity after five days of storage at 4 °C. Bordonein-L’s activity was higher than the control when stored in 2.8 % mannitol or 8.5 % sucrose.

**Conclusions:**

This research is pioneering in its isolation, characterization and enzyme stability evaluation of an LAAO from CdtV, denominated bordonein-L. These results are important because they increase the knowledge about stabilization of LAAOs, aiming to increase their shelf life. Since the maintenance of enzymatic activity after long periods of storage is essential to enable their biotechnological use as well as their functional studies.

## Background

L-amino acid oxidases (LAAOs) are enantioselective flavoenzymes that catalyze the stereospecific oxidative deamination of L-amino acids. An amino acid intermediate is hydrolyzed, releasing α-keto acids and ammonia. Concomitantly, the reduced non-covalently bond cofactor – flavin mononucleotide (FMN) or flavin adenine dinucleotide (FAD) – reoxidizes on molecular oxygen, producing hydrogen peroxide [1].

LAAOs are found in such diverse life forms as bacteria, marine organisms, fish, cyanobacteria, fungi, green algae, and snake venoms (SV) from the families Crotalidae, Elapidae and Viperidae [1–12].

SV-LAAOs are, in general, non-covalently bonded to FAD and their FAD-binding site shares sequential similarity with human monoamine oxidase, mouse interleukin 4-induced, bacterial and fungal LAAOs [1, 13]. SV-LAAOs usually constitute from 0.15 to 5 % of the snake venom protein, with some exceptions, such as the LAAO of *Bungarus caeruleus*, which represents 25 % of the total protein [14]. Several biological activities have been attributed to SV-LAAOs, including cytotoxicity, mild myonecrosis, apoptosis induction, induction and/or inhibition of platelet aggregation, as well as hemorrhagic, hemolytic, edematogenic, antibacterial, antiproliferative, antiparasitic and anti-HIV activities [14–25]. These activities are considered the result of the release of hydrogen peroxide, which produces oxidative stress [26]. However, the role of LAAOs in the venom has not been elucidated yet [26].

SV-LAAOs exhibit a wide range of isoelectric points (pI) from about 4.4 to 8.1, although it is unknown whether the different charges result in distinct pharmacological properties [13]. These enzymes prefer hydrophobic L-amino acids, because of substrate specificity related to side-chain binding sites [27].

LAAO activity is inhibited in the presence of ethylenediaminetetraacetic acid (EDTA), N-ethylmaleimide, phenylmethanesulfonyl fluoride (PMSF), glutathione and 1, 10-phenanthroline, since its cofactor is reduced under these conditions [14]. Furthermore, bivalent cations show different effects on LAAO activity. Manganese and calcium ions do not affect its specific activity. The LAAO from *C. adamanteus* requires Mg^2+^, while those from *Lachesis muta* and *Bothrops brazili* are inhibited by Zn^2+^ [14].

The cytotoxic effect of Bl-LAAO from *B. leucurus* venom was inhibited by about 25 % in the presence of catalase, an enzyme that cleaves hydrogen peroxide [17]. Additionally, the LAAOs of *Naja naja kaouthia* and *Calloselasma rhodostoma* venoms were inhibited by polyphenols from *Areca catechu* and *Quercus infectoria* extracts evaluated by *in vitro* tests [28]. Although the ethylacetate extract from *Azima tetracantha* leaves exerts an *in vitro* inhibitory activity on toxic enzymes from *B. caeruleus* and *Vipera russelli* venoms, LAAOs from neither venom was inhibited [29].

The LAAOs have shown maximum absorbance at 465 and 380 nm because of their bond with FAD [13]. Small changes in the absorption spectra of SV-LAAOs were observed after inactivation by freezing and thawing or modification of the ionic composition and pH conditions, indicating alterations in the microenvironment of the FAD cofactor [30]. Most of the studies in this area were published in the 1950s and 1960s [31–35]. One example is the inactivation of an LAAO isolated from *C. adamanteus* venom by high temperature and freezing. The higher the temperature or the pH of storage buffer, the higher the enzymatic inactivation, an inactivation that may be lower in the presence of chloride ions. On the other hand, at lower temperatures (freezing), the inactivation and storage buffer pH are inversely related. However, chloride ions were not able to prevent enzymatic inactivation in this case [31, 32]. Further studies showed that the inactivation of LAAOs causes changes in optical rotatory dispersion whereas the redox properties of free flavin are similar to those of the inactive enzyme [33, 35]. The change in redox properties suggests the loss of most interactions between flavin and apoprotein. Raibekas and Massey [36] extracted the cofactor of the LAAO from *C. adamanteus* venom at pH 3.5, rebound it at pH 8.5 and restored the enzymatic activity in the presence of 50 % glycerol followed by dialysis at 4 °C against 0.1 M Tris–HCl buffer, pH 7.5, containing 0.1 M KCl [36].

Due to their participation in metabolic pathways involving nitrogen and their antimicrobial, antiviral and antitumor effects, SV-LAAOs are considered a promising biotechnological agent and a tool for investigating cellular processes [13, 14]. However, diverse conditional factors that can reduce the stability of biocatalysts – including temperature, pH, oxidative stress, the solvent, binding of metal ions or cofactors, and the presence of surfactants – limit the industrial use of enzymes [37, 38]. Working under operational conditions of enzyme stability, the process costs are reduced [37], since the enzyme is active when in use and keeps active over time [39].

Two reports have shown that the presence of univalent ions or substrates for LAAOs and analogues of the prosthetic group (competitive inhibitors) prevents the inactivation of some SV-LAAOs [32, 40]. However, no additional studies have addressed the use of additives to maintain LAAO activity, which is highly desirable for industrial applications.

The use of additives to maintain proteins in their active forms is widespread throughout the pharmaceutical industry. For example, cyclodextrins are employed as excipients in pharmaceutical formulations in order to avoid protein aggregations to keep the protein in its active form [41]. There is a huge diversity of additives that act as cryoprotectants. Sugars and polyols, such as sucrose and mannitol, respectively, are used as protein stabilizers since they are able to interact with protein through hydrogen bonds to replace the protein-water molecular interactions [42, 43]. Amino acids are also used as cryoprotectants [43]. Usually, adjuvants are employed at a percentage that ranges from 0.5 to 2 %, although higher concentrations have already been tested [44–46].

Therefore, this study isolated an LAAO from *C. durissus terrificus* venom (CdtV), denominated bordonein-L, and evaluated the effect of different additives (mannitol, sucrose, L-Lys and L-Gly) as cryoprotectants for the enzyme.

## Methods

### Isolation of bordonein-L

Cdt yellow venom from the Ribeirão Preto region (21° 10′ 36″ S, 47° 49′ 15″ W) was obtained from specimens kept in the central snake house (University of São Paulo, Ribeirão Preto, SP, Brazil), in accordance with the guidelines of the Brazilian Institute of Environment and Renewable Natural Resources (IBAMA).

Desiccated CdtV (1 g) was purified through cation exchange chromatography, as described by Bordon et al*.* [47]. The CM5 fraction obtained in the first chromatographic step was fractionated on a HiPrep 16/60 Sephacryl S-100 HR column (1.6 × 60 cm, GE Healthcare, Sweden) equilibrated and eluted with 0.05 M sodium acetate buffer containing 0.15 M NaCl, pH 5.5, at a flow rate of 0.5 mL/min. The subfraction CM5S2 was applied on two 1-mL HiTrap Heparin HP columns (GE Healthcare) connected in a series equilibrated with 0.05 M sodium acetate buffer, pH 5.5. Adsorbed proteins were eluted using a step concentration gradient from 0 to 100 % of buffer B (1 M NaCl in the same buffer) at a 1.0 mL/min flow rate. To assess its purity degree, the peak H7 (LAAO bordonein-L) was submitted to RP-FPLC, as described by Bordon et al*.* [47].

### Determination of proteins

Total proteins were determined by the 280/205 nm absorption method [48].

### Determination of molecular mass

SDS-PAGE (10 %) was run according to the description of Laemmli [49]. The gel was stained with PlusOne Coomassie PhastGel Blue R-350 (GE Healthcare, Sweden) whereas periodic acid-Schiff (PAS) staining was employed to detect glycoproteins [50]. The hyaluronidase CdtHya1, a glycoprotein recently isolated from CdtV, was used as the control [47].

The molecular mass of bordonein-L was estimated by gel filtration chromatography on a Superdex 200 10/300GL column (GE Healthcare) calibrated with the following protein molecular mass standards: 12.4, 29, 66, 150 and 200 kDa (Sigma-Aldrich Co., United States). Blue dextran (2000 kDa, Sigma-Aldrich Co.) was used to determine the void volume. The column was equilibrated whereas the standards and the enzyme were eluted with the same buffer used on HiPrep 16/60 Sephacryl S-100 HR column. Each standard was filtered individually through the Superdex column and a calibration curve was constructed.

The molecular mass of bordonein-L was also analyzed by a MALDI-TOF mass spectrometer (Ultraflex II, Bruker Daltonics, Germany). MS spectrum was acquired in positive linear mode in the mass range 10,000-70,000 Da. TFA 0.1 % (10 μL) was added to the lyophilized enzyme. This solution was mixed (1:1) with sinapinic acid (20 mg/mL in 50/50 0.2 % ACN/TFA, v/v); and 2 μL of this mixture was spotted on a MALDI plate (384 positions) using the dried droplet method.

### Bordonein-L sequencing and *in silico* analysis

The N-terminal of bordonein-L was determined by Edman degradation in an automated protein sequencer model PPSQ-33A (Shimadzu Co., Japan) and compared with sequences deposited in the Basic Local Alignment Search Tool (BLAST) [51]. The alignment was created by MultAlin Interface Page [52] and the figure was generated by ESPript [53] server.

### LAAO activity

The LAAO activity of bordonein-L was performed through a microplate colorimetric assay according to modifications on the Kishimoto and Takahashi method [54]. Bordonein-L was incubated at 37 °C for 60 min with 0.002 M o-phenylenediamine (OPD) (Sigma-Aldrich Co.), 1 U/mL horseradish peroxidase (Sigma-Aldrich), 0.005 M L-Leucine (Sigma-Aldrich) and 0.05 M Tris–HCl buffer, pH 7.0. The reaction was stopped with 2 M H_2_SO_4_ and the absorbance was measured at 492/630 nm. LAAO activity was also evaluated at different pH levels (5.0-9.0).

### LAAO stability

The evaluation of LAAO stability was performed for 40 days at different concentration levels (1.4 %, 2.8 % and 8.5 %) of mannitol, sucrose, L-lysine and L-glycine, stored at 4 °C. Bordonein-L activity was also evaluated after being frozen (−20 °C) for a period of five days. The evaluation of enzymatic activity after lyophilization was performed as soon as this process was finished. The assays were carried out according to the LAAO activity assay previously described. Control consisted of bordonein-L in the absence of additives and storage at 4 °C. The enzyme was protected from light under all the tested conditions.

### Statistical analysis

LAAO activity data were expressed as mean ± standard error of mean (SEM). The analysis of variance (ANOVA) test was employed to evaluate data on LAAO activity in the presence of additives and to compare lyophilized, frozen and LAAO at 4 °C (five days), whereas the *t* test was utilized to compare LAAO stability after freezing versus already lyophilized. They were statistically significant when *p* < 0.05.

## Results

### Isolation of bordonein-L

Bordonein-L was purified in three chromatographic steps: cation exchange, molecular exclusion and affinity chromatography.

**Fig. 1:**
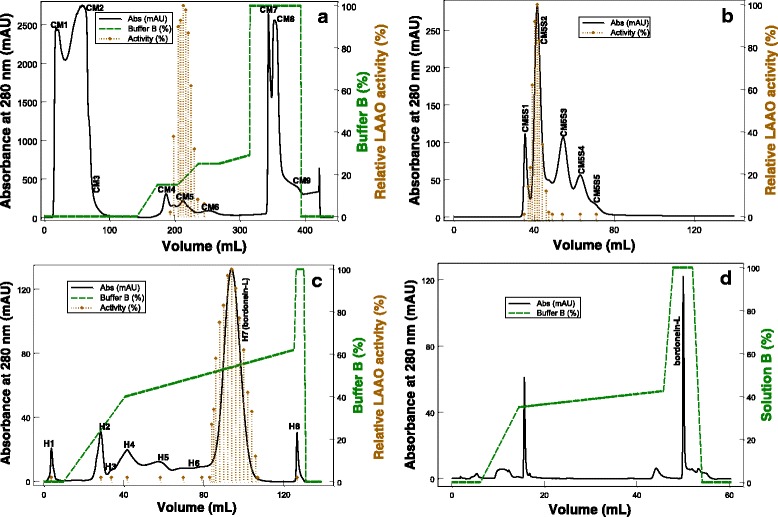
Isolation of Bordonein-L. Absorbance was monitored at 280 nm, at 25 °C, using a FPLC Äkta Purifier UPC-10 system. The dotted lines represent the concentration gradient. The vertical bars indicate the LAAO activity. **a** CdtV (1 g) was dispersed in 50 mL of 0.05 M sodium acetate buffer, pH 5.5 (buffer A) and the supernatant was fractionated on a CM-cellulose-52 column (1.0 × 40 cm) using a concentration gradient from 0 to 100 % of buffer B (1 M NaCl in buffer A). **b** The fraction CM5 was filtered on a HiPrep 16/60 Sephacryl S-100 HR column (1.6 × 60 cm) using 0.05 M sodium acetate buffer containing 0.15 M NaCl, pH 5.5. **c** Affinity chromatography of the CM5S2 fraction on HiTrap Heparin HP column (two 1-mL columns connected in series) using a concentration gradient from 0 to 100 % of buffer B. **d** Reversed-phase FPLC of H7 (bordonein-L) on a C4 column (0.46 × 25 cm, 5 μm particles) using a concentration gradient from 0 to 100 % of solution B (60 % acetonitrile in 0.1 % TFA)

LAAO activity was detected in the CM5 fraction (vertical bars, Fig. 1a) eluted from CM-cellulose-52 column. This fraction corresponds to 1.8 % of the total protein (Table 1). The CM5 fraction was applied on a HiPrep 16/60 Sephacryl S-100 HR column and LAAO activity was detected in the CM5S2 fraction (Fig. 1b), which was submitted to affinity chromatography on a HiTrap Heparin HP column. Thus, pure LAAO (peak H7), denominated bordonein-L, was obtained (Fig. 1c). The pure enzyme represents 48.3 % of the total activity and 0.5 % of the total protein of the venom (Table 1). Bordonein-L was then applied on a C4 column (Fig. 1d) and the main peak was submitted to Edman degradation.

**Table 1:** Specific activity and recovery of active fractions eluted during the purification procedure of bordonein-L

Fraction	Total protein (mg)^a^	Protein recovery (%)	One unit of LAAO activity (mg)^b^	Specific activity (U/mg)^c^	Total LAAO activity (U)	Yield (%)	Relative activity
CdtV	556.0	100.0	14.29	0.07	38.9	100.0	1.0
CM5	10.3	1.8	0.29	3.47	35.7	91.8	49.6
CM5S2	4.3	0.8	0.14	6.96	29.9	76.9	99.4
H7 (bordonein-L)	2.7	0.5	0.14	6.96	18.8	48.3	99.4

^a^Total protein quantified by absorbance method 280/205 nm (SCOPES, 1974)

^b^LAAO activity unit (U): amount of protein (mg) able to release 1.0 μmol of H_2_O_2_ per minute

^c^Specific activity: amount of H_2_O_2_ (μmol) released per minute per mg of protein

### Determination of molecular mass

**Fig. 2:**
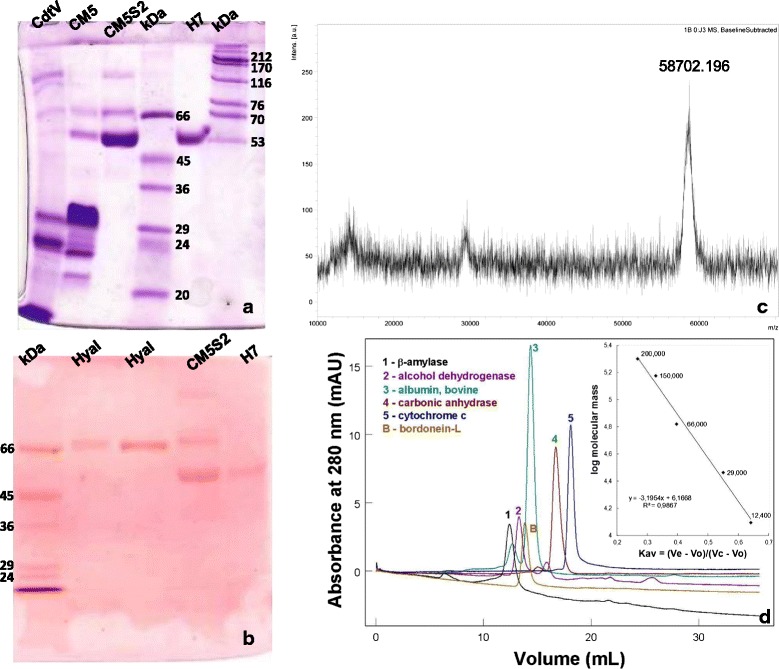
Determination of molecular mass. **a** SDS-PAGE (10 %) under non-reducing conditions stained with Coomassie Blue R-350. **b** SDS-PAGE (10 %) under non-reducing conditions stained with periodic acid-Schiff (PAS) to detect glycoprotein. Hyal: hyaluronidase CdtHya1 (glycoprotein control), H7: bordonein-L. **c** Mass spectrum of bordonein-L obtained by MALDI-TOF (positive linear mode). **d** Exclusion molecular of molecular mass standards and bordonein-L (20 μg/100 μL) on a Superdex 200 10/300GL (1 × 30 cm) column equilibrated and eluted with 0.05 M sodium acetate buffer containing 0.15 M NaCl, pH 5.5, at a flow rate of 0.5 mL/min. Insert: calibration curve of the Superdex 200 10/300GL column with molecular mass standards (12,400-200,000 Da)

SDS-PAGE under non-reducing conditions indicated that the peak H7 (bordonein-L) showed a high degree of purity while its monomer presented around 53 kDa (Fig. 2a), versus 56 kDa under reducing conditions (data not shown). Periodic acid-Schiff (PAS) staining evidenced that bordonein-L is a glycoprotein (Fig. 2b). The molecular mass of 58,702 Da was determined by MALDI-TOF (linear positive mode) mass spectrometry (Fig. 2c). Gel filtration under non-reducing conditions revealed a protein of approximately 101 kDa (Fig. 2d), indicating that bordonein-L is a dimer protein.

### *In silico* assays

**Fig. 3:**
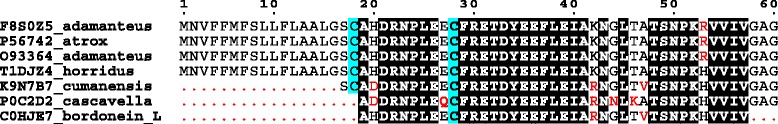
Multiple sequential alignment of snake venom L-amino acid oxidases from the genus *Crotalus*. Initial N-terminal of bordonein-L [Swiss-Prot: C0HJE7, bottom] and LAAOs from crotalic venoms: *C. adamanteus* [Swiss-Prot: F8S0Z5, O93364], *C. atrox* [Swiss-Prot: P56742], *C. horridus* [Swiss-Prot: T1DJZ4], *C. d. cumanensis* [Swiss-Prot: K9N7B7 – fragment] and *C. d. cascavella* [Swiss-Prot: P0C2D2 – fragment]. The highly conserved residues in bordonein-L are highlighted in black. The amino acid residues in red indicate low consensus. Cys residues are shaded in blue. The alignment and figure were generated by the servers MultAlin [52] and ESPript [53], respectively

The sequence of the first 39 N-terminal amino acid residues from bordonein-L was determined by Edman degradation and appears in the UniProt Knowledgebase under the accession number C0HJE7. This primary sequence exhibited high identity with other SV-LAAOs of the genus *Crotalus* (Fig. 3).

### LAAO activity and stability

**Fig. 4:**
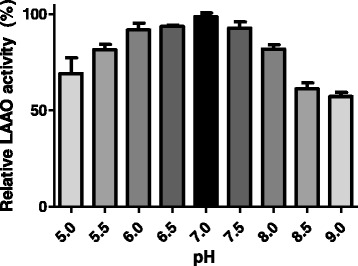
pH profile of LAAO activity. *Crotalus durissus terrificus* crude soluble venom, horseradish peroxidase, OPD and L-leucine were incubated in different 0.05 M buffers, at different pHs (5.0 to 9.0), for 60 min at 37 °C

**Fig. 5:**
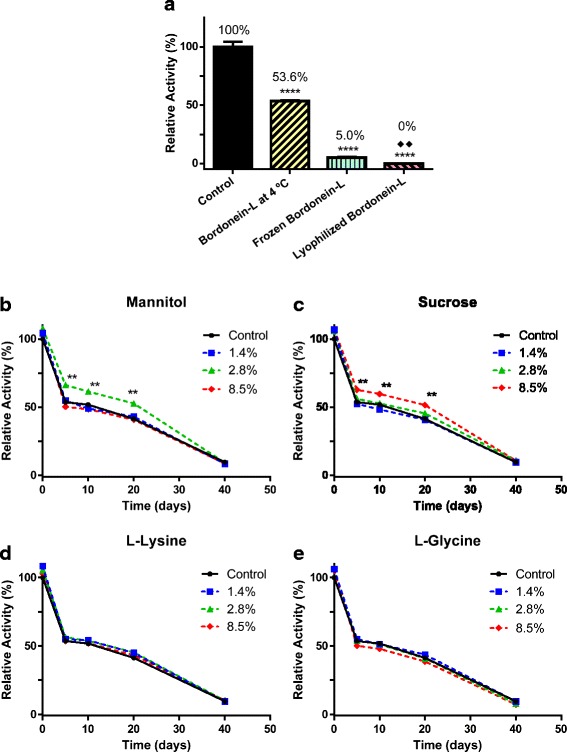
Bordonein-L stability. **a** Evaluation of stability after five days at −20 °C and 4 °C and as soon as the lyophilization was finished. The stability was also evaluated for 40 days in the presence of (**b**) mannitol, (**c**) sucrose, (**d**) L-lysine and (**e**) L-glycine. All samples were kept protected from light. Each point represents the mean ± S.E.M. (*n* = 3) at each additive concentration (**p < 0.0001 compared to the respective control using one-way ANOVA test). Each bar represents the mean ± S.E.M. (*n* = 3) at 4 °C, freezing and lyophilization conditions (****p < 0.0001 when 4 °C, freezing and lyophilization was compared to control and when freezing and lyophilization compared to 4 °C using one-way ANOVA test; ♦♦ *p* < 0.05 when freezing and lyophilization were compared to each other using the *t* test)

Bordonein-L showed an optimum pH of 7.0 (Fig. 4) and lost around 50 % of its activity in the first five days of storage at 4 °C (Fig. 5a-e). The frozen bordonein-L did not show enzymatic activity after lyophilization (Fig. 5a). Low activity (5 %) was also seen after thawing (Fig. 5a). Furthermore, LAAO activity was statistically significant when freezing and lyophilization were compared (Fig. 5a). L-lysine and L-glycine were not able to avoid the loss of activity at the tested concentrations (Fig. 5d and e). Bordonein-L activity was decreased when stored in 2.8 % mannitol, but during the course of the determined period of time (20 days), it was higher than the control. The enzymatic activity was the same as the control in the presence of other mannitol concentrations (1.4 % and 8.5 %) (Fig. 5b). On the other hand, 8.5 % sucrose kept bordonein-L more active than control during the first 20 days. Other tested sucrose concentrations were unable to keep bordonein-L more active than the control in the same time period (Fig. 5c).

## Discussion

There are 78 and 51 known primary sequences of SV-LAAOs deposited in the NCBI and UniProt databanks, respectively. However, the LAAO from *Crotalus durissus terrificus* venom (CdtV), one of the most studied snake venoms in Brazil, had not been assessed previously.

This is the first report of an LAAO from CdtV, denominated bordonein-L. The enzyme was isolated in three chromatographic steps and represented 0.5 % of the soluble venom protein. The specific activity for the soluble venom was 0.07 against 6.96 for bordonein-L, representing a 99.4-fold purification. The fractionation of 1 g of CdtV yielded only 2.7 mg of bordonein-L, a yield approximately four fold lower than the one obtained from the purification of 1 g of *C. adamanteus* venom [36]. However, its recovery is within the range from 0.15 to 5 % of the total protein observed in other snake venoms [14]. Significant differences in activity and protein concentration are observed even in snake venoms from the same species and region, as recently reported for the Cdt venom from the Botucatu region (SP, Brazil) [55].

Bordonein-L is a homodimeric glycoprotein. Molecular sieve chromatography under non-reducing conditions revealed a protein of approximately 101 kDa, while its mass was estimated at about 53 kDa by SDS-PAGE and 58,702 Da by mass spectrometry. SV-LAAOs are usually homodimeric FAD-binding glycoproteins with a molecular mass of around 110–150 kDa when measured by gel filtration under non-denaturing conditions and around 50–70 kDa when assayed by SDS-PAGE under reducing and non-reducing conditions [13]. Our results indicate that bordonein-L is a non-covalently associated homodimer, as reported for most SV-LAAOs.

The sequence of the first 39 N-terminal amino acid residues of bordonein-L exhibited identity with other SV-LAAOs, since the amino-terminal region is highly conserved. A high degree of similarity (>84 %) has been described among the primary sequences of SV-LAAOs even when comparing distinct genera [14].

Bordonein-L exhibited more than 80 % of relative activity in the pH range from 5.5 to 8.0, showing maximum activity at pH 7.0. Other SV-LAAOs show an active conformation at a pH ranging from 5.5 to 7.5, being inactivated at extremely basic pHs [34]. We observed an approximately 50 % loss of LAAO activity in the first five days of storage at 4 °C, almost complete inactivation after freezing and thawing, and total inactivation after lyophilization. The activity of the LAAO isolated from *C. adamanteus*, which shares high sequence identity with bordonein-L, is also greatly decreased by freezing [31, 32]. Other SV-LAAOs presented similar results [13]. Therefore, we suggest that bordonein-L be kept at 4 °C and near neutral pH to avoid its inactivation.

In relation to the stability of bordonein-L, L-glycine and L-lysine did not prevent the loss of enzymatic activity during the 40 days of storage at 4 °C, probably because they are not able to effectively interact with the active site in contrast to the hydrophobic L-amino acids and competitive inhibitors. L-glycine is the smallest amino acid and this small size may hinder its interaction with the catalytic site of bordonein-L. On the other hand, the amino acid L-lysine presents high polarity and the presence of polar groups might disrupt hydrophobic interactions. Hydrophobic L-amino acids, e.g. L-leucine, were not tested in this study as cryoprotectants because they are usually the preferred substrates of LAAOs whereas changes in the amino acid concentration would occur due to their concomitant oxidation during the activity assay, which would prevent the correct quantification of the LAAO activity [32].

Bordonein-L’s activity was higher than the control during the first 20 days when stored in 2.8 % mannitol or 8.5 % sucrose. At those concentrations, mannitol and sucrose interacted with bordonein-L through hydrogen bonds, which probably stabilized the enzyme by replacing the water molecular interactions, as reported for other proteins [42, 43]. However, after 40 days of storage, bordonein-L lost almost all of its activity even in the presence of additives. The rapid loss of activity (around 50 %) in the first five days and activity loss even in the presence of additives lead us to speculate that an alteration in the cofactor, such as oxidation or reduction, and/or changes in the catalytic site are responsible for the loss of LAAO activity since they may hinder the interaction among flavin, protein and substrate. The reduction of enzymatic activity as a result of FAD loss or conformational alterations was reported in other LAAOs [30, 33, 35]. Some conformational changes at the catalytic site were also suggested for gyroxin, another enzyme isolated from CdtV, whose catalytic efficiency was decreased in the presence of Mn^2+^ and Cu^2+^ [56].

The incorporation of additives to improve the stabilization of enzymes is the oldest and one of the most reliable enzyme stabilization methods, being employed in the most marketed enzyme formulations [57]. Since LAAOs are considered a promising biotechnological agent and a tool to investigate cellular processes, the retention of its enzymatic activity over time is essential [13, 14].

## Conclusions

An LAAO, denominated bordonein-L, was isolated from CdtV and presented higher enzymatic activity than the control when stored in 2.8 % mannitol or 8.5 % sucrose. These results may help the search for new additives to be used in stabilizing the LAAO, with the objective of increasing the shelf life of the enzyme.

## Abbreviations

ANOVA: Analysis of variance
BLAST: Basic local alignment search tool
CdtV: *Crotalus durissus terrificus* venom
EDTA: Ethylenediaminetetraacetic acid
FAD: Flavin adenine dinucleotide
FMN: Flavin mononucleotide
LAAO: L-amino acid oxidase
MALDI-TOF: Matrix assisted laser desorption ionization time of flight
OPD: O-phenylenediamine
PAS: Periodic acid-Schiff
pI: Isoelectric point
PMSF: Phenylmethanesulfonyl fluoride
RP-FPLC: Reversed-phase fast protein liquid chromatography
SDS-PAGE: Sodium dodecyl sulphate polyacrylamide gel electrophoresis
SEM: Standard error of mean
SV: Snake venom.
